# Prior event rate ratio adjustment for hidden confounding in observational studies of treatment effectiveness: a pairwise Cox likelihood approach

**DOI:** 10.1002/sim.7051

**Published:** 2016-08-01

**Authors:** Nan Xuan Lin, William Edward Henley

**Affiliations:** ^1^ Department of Mathematics and Information SciencesNorthumbria UniversityNewcastle upon TyneNE2 1XEU.K.; ^2^ Health Statistics Group, Institute of Health ResearchUniversity of Exeter Medical SchoolExeterEX1 2LUU.K.

**Keywords:** prior event rate ratio, pairwise Cox model, unmeasured confounding, observational study, treatment effect

## Abstract

Observational studies provide a rich source of information for assessing effectiveness of treatment interventions in many situations where it is not ethical or practical to perform randomized controlled trials. However, such studies are prone to bias from hidden (unmeasured) confounding. A promising approach to identifying and reducing the impact of unmeasured confounding is prior event rate ratio (PERR) adjustment, a quasi‐experimental analytic method proposed in the context of electronic medical record database studies. In this paper, we present a statistical framework for using a pairwise approach to PERR adjustment that removes bias inherent in the original PERR method. A flexible pairwise Cox likelihood function is derived and used to demonstrate the consistency of the simple and convenient alternative PERR (PERR‐ALT) estimator. We show how to estimate standard errors and confidence intervals for treatment effect estimates based on the observed information and provide R code to illustrate how to implement the method. Assumptions required for the pairwise approach (as well as PERR) are clarified, and the consequences of model misspecification are explored. Our results confirm the need for researchers to consider carefully the suitability of the method in the context of each problem. Extensions of the pairwise likelihood to more complex designs involving time‐varying covariates or more than two periods are considered. We illustrate the application of the method using data from a longitudinal cohort study of enzyme replacement therapy for lysosomal storage disorders. © 2016 The Authors. *Statistics in Medicine* Published by John Wiley & Sons Ltd.

## 1 Introduction

Observational studies, based on routinely collected patient data or data from population‐based cohorts, offer a rich source of information for evaluating real‐world effectiveness of medical treatments 1. With the anticipated growth in implementation of electronic health record systems, there is increasing scope for using large observational datasets to inform the design of randomized trials and to address clinical questions for which trials are unlikely to be conducted because of ethical or logistical considerations. However, a major challenge in adopting this approach is the need to remove bias introduced due to confounding by indication or other biases due to the effect of unmeasured covariates 2. Analyses that fail to account for relevant confounders may have important negative consequences for health policy and patient safety.

Tannen *et al*. 3 introduced prior event rate ratio (PERR) adjustment, a new quasi‐experimental analytic approach to identifying and reducing hidden (unmeasured) confounding in the analysis of time‐to‐event data from clinical database studies.

**Figure 1:**
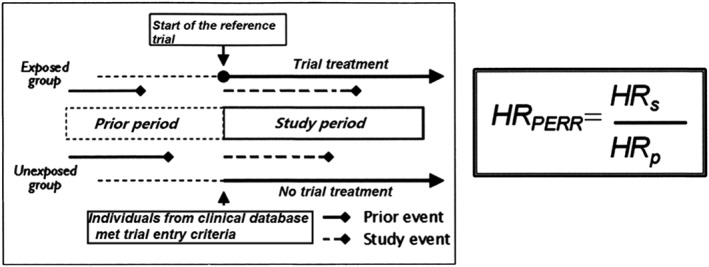
Schematic for the prior event rate ratio adjustment method.

The PERR approach replicates a randomized trial by identifying a group of individuals from a clinical database using the same inclusion and exclusion criteria, a similar study time frame and a similar treatment regimen as the trial. The exposed group is the individuals who received treatment within the recruitment interval of the trial. The unexposed group are individuals not receiving the treatment within the recruitment window. Previous studies using the PERR method have generally matched unexposed patients to exposed patients on an index date (often, the date that the exposed patients first received treatment). The method relies on a before‐and‐after design and assumes that the hazard ratio of the exposed to unexposed for a specific outcome before the start of the trial reflects the combined effect of all confounders independent of any influence of treatment. Let *H* *R*_*s*_ be the unadjusted hazard ratio of treated versus control during the study from a Cox regression model, and *H* *R*_*p*_ be the corresponding unadjusted hazard ratio of treated versus control in the prior period. To account for unmeasured confounding, the PERR‐adjusted hazard ratio is given by *H* *R*_*PERR*_=*H* *R*_*s*_/*H* *R*_*p*_. The schematic of this method is displayed in Figure 1.

Two studies by Tannen and colleagues reported good concordance between PERR results and those from published RCTs. First, observational data from the clinical practice research datalink were used to replicate the Scandinavian Simvastatin Survival Study of statin treatment for hypercholesterolemic subjects with coronary heart disease 4; second, data from the same source were used to replicate two randomized trials of angiotensin‐converting enzyme inhibitors in patients without congestive heart failure at high risk for cardiovascular disease 5. Tannen *et al*. 5 conducted a broader validation study in which the PERR adjusted *HR*s were not significantly different from the trial *HR*s in five out of eight comparisons of cardiovascular outcomes where unadjusted *HR*s were inconsistent with the trial results (suggesting the presence of unmeasured confounding). However, Yu *et al*. 6 noted that the original PERR method frequently led to attenuated treatment estimates in simulation experiments and introduced an alternative, referred to as PERR‐ALT, based on a paired Cox model. This approach gave unbiased estimates in simulation studies where the unmeasured confounder effect did not vary temporally. More generally, both PERR methods performed well in reducing bias when the treatment effect was large compared with any confounder‐treatment interaction. The original PERR estimator was more computationally stable than PERR‐ALT when the event rate was low and the sample size was limited.

Given the promising results in these early studies, the PERR method is gaining acceptance as a useful approach for addressing hidden confounding when comparing or evaluating treatments in observational studies 2. For example, PERR adjustment has been used to assess the incidence of *Campylobacter* and *Salmonella* infection in patients‐prescribed proton pump inhibitors compared with controls, using electronic health records from the secure anonymized information linkage databank 7. Tannen *et al*. 8 proposed a strategy for performing comparative effectiveness research using the THIN database in which PERR adjustment is used to remove bias because of unmeasured confounding.

The aim of this paper is to set out a detailed statistical framework for using a pairwise approach to PERR adjustment and to address some of the methodological challenges. Yu *et al*. 6 present a simple and convenient formula for PERR‐ALT adjustment. We extend their approach by deriving a flexible pairwise Cox likelihood function and using this to show that the PERR‐ALT method is consistent, under relevant assumptions. The pairwise likelihood can be used to obtain consistent estimates of treatment effects, other measured covariates, and period effects. We also consider ways in which the likelihood can be extended to relax the assumptions of the original PERR method. We address the issue of estimating standard errors and confidence intervals for the treatment effect estimates. Previous work has used a bootstrapping technique to estimate confidence intervals for the PERR‐ALT method because of difficulties in estimating the covariance between *H* *R*_*s*_ and *H* *R*_*p*_ 8. We provide a direct approach based on the observed information matrix.

The paper is organized as follows. The definitions of prior and study event times, the hazard models, and assumptions required for using the pairwise Cox method (as well as PERR and PERR‐ALT ) in the analysis of observational data are clarified in Section 2. In Section 3, we consider the nature of the bias in the original PERR method using asymptotic bias formulae. In Section 4, we propose a statistical test for detecting hidden confounding using prior event data. The pairwise Cox likelihood function is derived in Section 5 and formulae given for estimating standard errors. We also consider how the method could be applied in the context of crossover trials. Section 6 discusses situations in which the PERR method will be prone to bias. Extensions of the pairwise likelihood that permit more flexible modeling are considered in Section 7. The method is applied to data from a longitudinal cohort study of enzyme replacement therapy for lysosomal storage disorders in Section 8.

## 2 Definitions, assumptions, and hazard model

We start by introducing some notation and setting out a general framework for quasi‐experimental analytic studies using a pairwise Cox approach. Here, random variables are denoted by upper case letters, and their values are denoted by lower case letters.

In what follows, we assume that there are two types of events of interest: the study event and the prior event. Usually, the study event is defined so that some individuals receive a treatment or exposure during or before the time of the study event (the exposed group) and some do not (the unexposed group). The prior event refers to an earlier event when no individuals are treated. However, these requirements are not compulsory. As illustrated later in an example related to treatment for diabetic retinopathy, there could be no order between the prior and study events, and patients can be treated during or before the time of the prior event. We note that the approach can easily be generalized to more than two events as considered in Section 7.2.

Designing studies based on the pairwise Cox likelihood method requires the user to define the events and time origins carefully to reflect the requirements of the research problem. First, the prior and study events need to be defined. Usually, the prior and study events should be of the same nature so that the unknown confounders will have the same effects on the hazards of prior and study events. For example, this may be a reasonable assumption in a study investigating the protective effects of estrogen replacement therapy on risk of myocardial infarction (MI) in post‐menopausal women where previous MI is used as the prior event 8.

In order to define the event times used in the statistical analysis, the time origins when individuals first become at risk for the prior and study events need to be defined in the context of the research problem. This fixes the prior and study periods that provide the framework for the pairwise design. We define $T_{p}^{*}$ for the variable of prior event time measured from the prior start point to a prior event and $T_{p}^{+}$ to be the corresponding variable for the prior censoring time. The prior start point is defined as the time origin when the individual is considered to be at risk of experiencing a prior event. The variable for the observed prior time is $T_{p}=\min\left(T_{p}^{*},T_{p}^{+}\right)$ with the variable for the prior censoring indicator $\Delta_{p}=I\left(T_{p}^{*}\leq T_{p}^{+}\right)$, where $I\left(T_{p}^{*}\leq T_{p}^{+}\right)$ is an indicator function such that $\Delta_{p}=I\left(T_{p}^{*}\leq T_{p}^{+}\right)=1$ if $T_{p}^{*}\leq T_{p}^{+}$ and 0 otherwise. We call the time span that the individual is at the risk of a prior event (or being observed for a prior event) the prior period, which is measured from the prior start point to the observed time *T*_*p*_. The definition of prior period means that an individual is at risk of a prior event (or being observed for a prior event) until the prior event is observed $\left(T_{p}=T_{p}^{*}\right)$ or right censored $\left(T_{p}=T_{p}^{+}\right)$. Similar definitions apply to the study start point, study period, and the variables $\left(T_{s}^{*},T_{s}^{+},T_{s},\Delta_{s}\right)$ for the study event, censoring and observed times, and censoring indicator, respectively. Note that it is generally not appropriate when using the pairwise and PERR methods to measure *T*_*s*_ from the time of receiving treatment as this is often not the time when an individual starts to be under the risk of a study event (see later).

**Figure 2:**
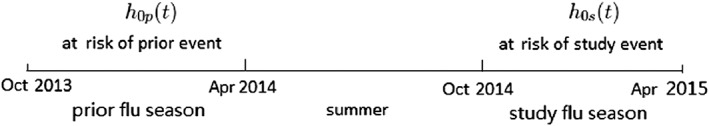
Timeline for an illustrative study of influenza vaccination effectiveness using the pairwise Cox method.

To illustrate the process of model formulation, we consider the example of assessing the effectiveness of influenza vaccination in older adults in the UK using electronic medical record data. Influenza is a major cause of illness and death amongst the elderly, and reliable estimates of vaccine effectiveness are important for informed vaccine policies and programs 9. To apply the pairwise Cox method, we first need to define the prior and study events: If the focus is on more serious outcomes, then one possible event would be hospitalization for an influenza‐related illness. We make the simplifying assumption that individuals are at risk of influenza during the winter season (from October 1 to March 31 each year) but not during the summer season (from April 1 to September 30 each year). Suppose data are available from the 2013/2014 and 2014/2015 influenza seasons. The prior and study time origins are October 1, 2013 and October 1, 2014, respectively (Figure 2).

Let *X* = (*X*_1_,…,*X*_*q*_)^*tr*^ be *q*‐measured covariates in the study period, where ^*tr*^ is the notation of transpose and *C* be the column vector for hidden covariates (or confounders). Conditional on *X* and *C*, (*T*_*p*_,*T*_*s*_) and $\left(T_{p}^{+},T_{s}^{+}\right)$ are assumed to be independent. The independent censoring assumption is sensible and has been widely used in multiple events models such as Prentice, Williams, and Peterson 10 and Wei, Lin, and Weissfeld 11 (hereafter, referred to as PWP and WLW). For example, in the influenza vaccination study, if patients are not infected with flu from October 2013 to April 2015, the prior and study events are right censored with $T_{p}^{+}=T_{s}^{+}=6$ months (i.e., the length of flu season). In this case, the censoring is similar to administrative censoring.

In the presence of death, if death is related to the prior or study events, a competing risk model will be needed 12. In this paper, we focus on the case where death is not related to the prior or study events and can be regarded as right censoring. If the individual died after the prior event and before the study period, *T*_*si*_=0 and Δ_*si*_=0. We will show that this kind of data has no contribution to the pairwise Cox likelihood.

Let $\left(T_{\mathrm{pi}},T_{\mathrm{pi}}^{*},T_{\mathrm{pi}}^{+},\Delta_{\mathrm{pi}},T_{\mathrm{si}},T_{\mathrm{si}}^{*},T_{\mathrm{si}}^{+},\Delta_{\mathrm{si}},X_{i},C_{i}\right)$ be *n*‐independent replicates of $\left(T_{p},T_{p}^{*},T_{p}^{+},\Delta_{p},T_{s},T_{s}^{*},T_{s}^{+},\Delta_{s},X,C\right)$. It is assumed that the hazard of a prior event at $T_{\mathrm{pi}}^{*}=t$ is as follows:

(1) $$h_{\mathrm{pi}}(t)=I\left(t\leq T_{\mathrm{pi}}\right)h_{0p}(t)\exp(\beta C_{i}),$$

where *h*_0*p*_(*t*) is the unspecified baseline hazard for the prior event, *β* is the vector of coefficients for *C*_*i*_, and *I*(*t*≤*T*_*pi*_) indicates whether *t* is within the prior period, that is, whether the individual is at the risk of a prior event (or being observed for a prior event) until the prior event is observed or a censorship occurs.

Similarly, we assumed the hazard of a study event at $T_{\mathrm{si}}^{*}=t$ is as follows:

(2) $$h_{\mathrm{si}}(t)=I\left(t\leq T_{\mathrm{si}}\right)h_{0s}(t)\exp\left(\theta X_{i}+\beta C_{i}\right),$$

where *h*_0*s*_(*t*) is the unspecified baseline hazard for the study event, *θ* = (*θ*_1_,…,*θ*_*q*_) is the vector of coefficients for *X*_*i*_, and *I*(*t*≤*T*_*si*_) is the indicator for the study period.

In (1) and (2), *β* *C*_*i*_ is assumed to be time invariant. In practice, the prior and study events need to be carefully defined to satisfy this assumption. As we said, usually the prior and study events should be of the same nature. As a consequence, the pairwise Cox likelihood approach is only applicable to problems where the prior and study events are non‐terminal events. In particular, the method cannot be applied when death is an outcome of interest.

We maintain that a careful clarification of research purpose is needed before one can choose a statistical model. Like the PWP and WLW models, the basic models (1)–(2) allow for the different events (prior and study) to have different baseline hazards (*h*_0*p*_ and *h*_0*s*_). The WLW model usually operates with a common time origin, while the basic models (1)–(2) allow the different event times to have different time origins. In some cases, the Andersen and Gill model is suitable if the events shared a common baseline hazard, while in other cases, it is reasonable to assume different baseline hazards for different events. For example, in the study of influenza vaccination, it is reasonable to allow different baseline hazards, *h*_0*p*_(*t*) and *h*_0*s*_(*t*), for the prior and study events because of the unknown changes in conditions between the two flu seasons (e.g., due to differences in the circulating strains of influenza).

The basic models (1)–(2) can be used wherever the PWP, WLW, and the Andersen and Gill (AG) 13 models are applicable. For example, if the prior and study events are the first and second events, respectively, then *T*_*pi*_ and *T*_*si*_ are the times for the first and second events, respectively. In this case, by adopting a time‐varying treatment indicator *X*_*i*_(*t*), the basic models (1)–(2) are the same as follows:

- the PWP model, if *T*_*si*_ is measured from the time point of the prior event;
- the WLW model, if *T*_*si*_ is measured from the same time origin as *T*_*pi*_; and
- the Andersen and Gill model, if *T*_*si*_ is measured from the same time origin of *T*_*pi*_ and we assume *h*_0_=*h*_0*p*_=*h*_0*s*_.

The definition of events and measurement of times are important when using the PERR/pairwise method. Returning to the estrogen replacement therapy example, it is not appropriate to define the prior event as the first MI (no matter whether the patient is treated or not) and the study event as the first MI after the treatment, and measure *T*_*s*_ from *τ*_*i*_, the treatment time for the *i*th individual. Consider, for example, if the treatment has no effect (*θ* = 0), a first MI after treatment at *T*_*si*_=*t* without a prior MI can be regarded as both the prior and study events and will consequently have two different hazards *h*_0*p*_(*τ*_*i*_+*t*) exp(*β* *c*_*i*_) ≠ *h*_0*s*_(*t*) exp(*β* *c*_*i*_) (the common baseline hazard *h*_0*s*_ could not be the same as *h*_0*p*_(*τ*_*i*_+*t*), which depends on individual *i*). For this example, it is more appropriate to define the prior event as the first MI and the study event as the second MI. The time *T*_*s*_ can be measured from the first MI (like the PWP model) or from the prior start point (like the AG and WLW models). The pairwise approach can be easily generalized to the cases where the third, fourth, or more MIs are of interest by using a time‐varying treatment variable.

Another example is an observational study of the effectiveness of laser photocoagulation in delaying the onset of blindness in patients with diabetic retinopathy. A patient could potentially experience blindness in both eyes; we define the prior and study events to be left and right eye blindness, respectively. In this example, there is no order between the prior and study events, and the prior and study periods overlap. The right eye can be blind before the left eye. The patient starts to be at the risk of blindness for both eyes from the same time origin. A WLW‐type model can be used:

$$\begin{array}{cc} h_{\mathrm{pi}}(t) & =I\left(t\leq T_{\mathrm{pi}}\right)h_{p0}(t)\exp\left(\theta_{p}X_{\mathrm{ip}}+\beta C_{i}\right)\\ h_{\mathrm{si}}(t) & =I\left(t\leq T_{\mathrm{si}}\right)h_{s0}(t)\exp\left(\theta_{s}X_{\mathrm{is}}+\beta C_{i}\right), \end{array}$$

where *X*_*ip*_=1 if the left eye was on treatment and 0 otherwise, *X*_*is*_=1 if the right eye was on treatment and 0 otherwise, and *θ*_*p*_ and *θ*_*s*_ are the treatment effect on the left and right eyes, respectively. The process of observing the left eye blindness is stopped (i.e., *I*(*t*≤*T*_*pi*_) = 0) if the left eye is blind $\left(t=t_{\mathrm{pi}}^{*}\right)$ or the follow‐up ends $\left(t=t_{\mathrm{pi}}^{+}\right)$. The likelihood function for this example is given in ((16)).

For the influenza vaccination example, the basic models (1)–(2) can be extended as follows:

$$\begin{array}{cc} h_{\mathrm{pi}}(t) & =I\left(t\leq T_{\mathrm{pi}}\right)h_{p0}(t)\exp\left(\theta X_{\mathrm{ip}}(t)+\beta C_{i}\right)\\ h_{\mathrm{si}}(t) & =I\left(t\leq T_{\mathrm{si}}\right)h_{s0}(t)\exp\left(\theta X_{\mathrm{is}}(t)+\beta C_{i}\right), \end{array}$$

where *T*_*pi*_ and *T*_*si*_ are respectively measured from October of 2013 and 2014 to the times of hospitalizations for an influenza‐related illness or to April of 2014 and 2015 (if the times are censored). The time origins are defined as the start of the 2013 and 2014 influenza seasons because these are the points at which patients first become at risk for a prior or study event, respectively. We note that in this example, there is a gap in being under risk between the prior and study periods (during the summer period in 2014). In this type of problem, it is not appropriate to define the study time origin at the first event (like the PWP model) or at the start of the prior period (like the AG and WLW models). The definition of the time origins in this example requires the indicators of vaccinations *X*_*ip*_(*t*) and *X*_*is*_(*t*) to be time‐varying: *X*_*ip*_(*t*) = 1 if the individual *i* was vaccinated at or before the time *t*_*pi*_=*t* and 0 otherwise; *X*_*is*_(*t*) = 1 if the individual *i* was vaccinated at or before the time *t*_*si*_=*t* and 0 otherwise. If there is a prior event at time $t_{\mathrm{pi}}^{*}$, then $T_{\mathrm{pi}}=t_{\mathrm{pi}}^{*}$. The indicator for the prior period *I*(*t*≤*T*_*pi*_) = 0 for $t>t_{\mathrm{pi}}^{*}$ reflecting the assumption that there is no risk of flu after a previous hospitalization related to influenza within the same flu season. This is because an individual contracting influenza develops antibodies to the circulating strain. If there is no prior event, the prior event time is censored, and the observed prior time *T*_*pi*_ is equal to the length of the flu season, that is, 6months. The indicator for the prior period *I*(*t*≤*T*_*pi*_) = 0 for *t* > 6 reflecting the assumption that there is no risk of flu after the flu season.

## 3 Bias in the prior event rate ratio method

Suppose the true hazards are (1) and (2). The PERR method fits the following models for the prior and study event times:

(3) $$I\left(t\leq T_{\mathrm{pi}}\right)h_{0p}^{*}(t)\exp\left(\theta_{p}^{*}X_{i}\right),$$

(4) $$I\left(t\leq T_{\mathrm{si}}\right)h_{0s}^{*}(t)\exp\left(\theta_{s}^{*}X_{i}\right),$$

respectively. The parameters $\theta_{p}^{*}=\log(HR_{p})$ and $\theta_{s}^{*}=\log(HR_{s})$ are the log‐hazard ratios without adjustment for *C*_*i*_ for prior and study events, respectively. The PERR‐adjusted log‐*HR* is given by $\log(HR_{\text{PERR}})=\theta_{\text{PERR}}=\theta_{s}^{*}-\theta_{p}^{*}$. The parameters $\theta_{p}^{*}$ and $\theta_{s}^{*}$ are estimated from the log partial likelihoods:

(5) $$\frac{1}{n}\sum_{i}^{n}\Delta_{\mathrm{ip}}\left\{\theta_{p}^{*}X_{i}-\log\sum_{j}^{n}I(T_{\mathrm{pi}}\leq T_{\mathrm{pj}})\exp\left(\theta_{p}^{*}X_{j}\right)\right\},$$

(6) $$\frac{1}{n}\sum_{i}^{n}\Delta_{\mathrm{is}}\left\{\theta_{s}^{*}X_{i}-\log\sum_{j}^{n}I(T_{\mathrm{si}}\leq T_{\mathrm{sj}})\exp\left(\theta_{s}^{*}X_{j}\right)\right\},$$

respectively.

However, the PERR adjustment cannot entirely remove the bias from the estimate of the treatment effect, that is, *θ*_*PERR*_≠*θ*. Lin *et al*. 14 studied the problem of hidden confounding in the Cox regression model and showed that due to the nonlinearity of the Cox model, the resulting bias is a complicated combination of three sources: (i) bias due to omission of hidden covariates, even if they are not confounders; (ii) bias due to censoring; and (iii) bias due to the hidden covariates being confounders. Here, we conducted a simple simulation study to illustrate the impact of these three sources of bias on the performance of the PERR method. We generated 100,000 paired times (*t*_*p*_,*t*_*s*_) for prior and study events from the models (1) and (2), respectively, with *h*_0*p*_(*t*) = *θ* = 1, *h*_0*s*_(*t*)= exp(1), *X* ∼ *Bin*(1,0.5) and the log *HR* of the hidden covariate, *β*, taking 100 sequenced values from −10 to 10. The data were fitted by the models (3) and (4). Because the sample size is large (*n* = 100,000), the bias of the unadjusted estimate ${\hat{\theta }}_{s}^{*}$ for the naive Cox model and the bias of the PERR estimate ${\hat{\theta }}_{\text{PERR}}$ were approximated by ${\hat{\theta }}_{s}^{*}-\theta$ and ${\hat{\theta }}_{s}^{*}-{\hat{\theta }}_{p}^{*}-\theta$, respectively.

**Figure 3:**
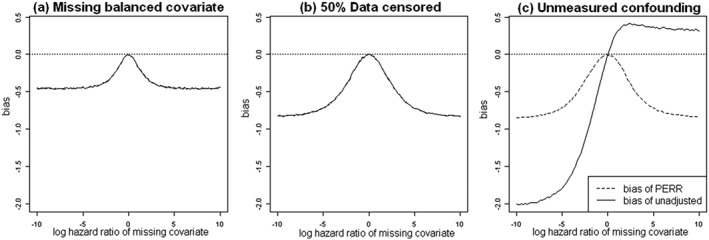
Bias curves for the prior event rate ratio (PERR) estimate *θ*_*PERR*_ and the unadjusted estimate *θ* ^∗^ of the naive Cox model in the presence of omitted covariates. Graphs are based on simulated dataset with *n* = 100,000 and true log‐hazard ratio of treatment *θ* = 1. An exponential baseline hazard was used with a rate parameter of *h*_0_(*t*) = 1. The censoring mechanism was uniform.

We present bias curves in Figure 3 showing the relationship between the bias of the PERR estimate and the value of *β* under three different scenarios to separate out the effect of the three potential sources of bias: Figure 3(a) shows the bias when there is a hidden balanced covariate *C* ∼ *Bin*(1,0.5) in the absence of censoring; Figure 3(b) shows the bias when again *C* ∼ *Bin*(1,0.5), but in addition, both *t*_*p*_ and *t*_*s*_ are 50*%* censored; Figure 3(c) shows the bias when again *t*_*p*_ and *t*_*s*_ are 50*%* censored, but *C* is now a hidden confounder with distribution *B* *i* *n*(1,0.3 + 0.4*X*) (i.e., the omitted covariate has the same marginal distribution as Figure 3(a) and (b), but the conditional distribution depends on *X*).

The three figures show that PERR adjustment is biased even if the hidden covariate *C* is not a confounder. In Figure 3(a) and (b), it is hard to see the difference between the PERR method and the naive unadjusted Cox model because the bias curves for $\theta_{s}^{*}$ and *θ*_*PERR*_ overlap closely for all values of *β*: PERR adjustment fails to remove any of the bias attributable to censoring or to hidden covariates that are balanced across treatment groups. The figures show that the bias of PERR adjustment increases with the absolute value of the log‐hazard ratio *β* of the hidden covariate. The direction of the bias is always the same: Treatment effect estimates are attenuated. The PERR adjustment is only successful in removing the bias attributable to confounding, as shown in Figure 3(c). The unadjusted Cox model fails to remove the hidden confounding bias, and the direction of the bias depends on the sign of *β* and the distribution of *C* conditional on *X*.

Although the models (3) and (4) are misspecified, the maximal likelihood estimates ${\hat{\theta }}_{p}^{*}$ and ${\hat{\theta }}_{s}^{*}$ converge to well‐defined constants as *n*→*∞* 15. The limiting values of ${\hat{\theta }}_{p}^{*}$ and ${\hat{\theta }}_{s}^{*}$ are sometimes called the ‘least false’ value 16. For simplicity of notation, we use the same $\theta_{p}^{*}$ and $\theta_{s}^{*}$ to denote these limits. By calculating the limits of the score functions of the likelihood ((5)) and ((6)) as *n*→*∞* under the true models (1) and (2) and following the result of Lin *et al*. 14, it can be shown that the limits $\theta_{p}^{*}$ and $\theta_{s}^{*}$ are the solutions of the equations:

(7) $$\begin{array}{cc} 0 & =U_{p}\left(\theta_{p}^{*}\right)=E\left(\Delta_{p}\left[X-\frac{E_{\mathrm{xc}}\left\{S_{p}^{+}(T_{p}|X)e^{\theta_{p}^{*}X}e^{-H_{0p}(T_{p})e^{\mathrm{\beta C}}}X\right\}}{E_{\mathrm{xc}}\left\{S_{p}^{+}(T_{p}|X)e^{\theta_{p}^{*}X}e^{-H_{0p}(T_{p})e^{\mathrm{\beta C}}}\right\}}\right]\right)\\ 0 & =U_{s}\left(\theta_{s}^{*}\right)=E\left(\Delta_{s}\left[X-\frac{E_{\mathrm{xc}}\left\{S_{s}^{+}(T_{s}|X)e^{\theta_{s}^{*}X}e^{-H_{0s}(T_{s})e^{\mathrm{\theta X}+\mathrm{\beta C}}}X\right\}}{E_{\mathrm{xc}}\left\{S_{s}^{+}(T_{s}|X)e^{\theta_{s}^{*}X}e^{-H_{0s}(T_{s})e^{\mathrm{\theta X}+\mathrm{\beta C}}}\right\}}\right]\right), \end{array}$$

where $H_{0p}(T_{p})=\int_{0}^{T_{p}}h_{0p}(t)\mathrm{dt}$, $H_{0s}(T_{s})=\int_{0}^{T_{s}}h_{0s}(t)\mathrm{dt}$, *E*_*xc*_ is the expectation with respect to *X* and *C*, and $S_{p}^{+}(T_{p}|X)$ and $S_{s}^{+}(T_{s}|X)$ are the survival functions of the prior and study censoring times conditional on *X*, respectively.

The first‐order Taylor series approximation for the bias of PERR adjustment is as follows:

$$\theta_{\text{PERR}}-\theta \approx I_{s}^{-1}(\theta )U_{s}(\theta )-I_{p}^{-1}(0)U_{p}(0),$$

where $I_{s}\left(\theta_{s}^{*}\right)=-\partial U_{s}\left(\theta_{s}^{*}\right)/\partial \theta_{s}^{*}$ and $I_{p}\left(\theta_{p}^{*}\right)=-\partial U_{p}\left(\theta_{p}^{*}\right)/\partial \theta_{p}^{*}$ .

## 4 Use of prior event data to detect unmeasured confounding

A hypothesis test for detecting unmeasured confounding can be developed based on the first equation in ((7)). Suppose *C*⊥*X* so that *C* is an unmeasured balanced covariate but not a confounder, the first equation in ((7)) reduces to the following:

(8) $$E(\Delta_{p}X)=E\left(\Delta_{p}\left[\frac{E_{x}\left\{S_{p}^{+}(T_{p}|X)e^{\theta_{p}^{*}X}X\right\}}{E_{x}\left\{S_{p}^{+}(T_{p}|X)e^{\theta_{p}^{*}X}\right\}}\right]\right).$$

Under the independent censoring assumption $T_{p}^{*}⊥T_{p}^{+}|(X,C)$ 11, the survival function for the prior censoring time can be written as $S_{p}^{+}(t|X,C)$. Because *X* is the set of covariates associated with the study period, $S_{p}^{+}(t|X,C)=S_{p}^{+}(t|C)$. With *C*⊥*X*, it follows:

(9) $$S_{p}^{+}(t|X)=\int S_{p}^{+}(t|X,c)f(c|X)\mathrm{dc}=\int S_{p}^{+}(t|c)f(c)\mathrm{dc}=S_{p}^{+}(t).$$

Therefore, ((8)) is further simplified to the following:

$$E(X)=\frac{E\left(e^{\theta_{p}^{*}X}X\right)}{E\left(e^{\theta_{p}^{*}X}\right)},$$

which has the solution $\theta_{p}^{*}=0$.

This provides the basis for the following procedure to test for the presence of an unmeasured confounder using the prior event data:

1. Suppose the null hypothesis is
  $$H_{0}:C⊥X\;\text{so that}\;\text{C}\;\text{is an unmeasured balanced covariate but not a confounder}.$$
2. To assess (9), fit the model (3) to the data *t*_*p*_ and *x* but with the censoring indicators reversed (i.e., by considering events as ‘censored’ observations and censored observations as ‘events’). If the *p*‐value for the estimate ${\hat{\theta }}_{p}^{*}$ is greater than 0.05, it indicates that (9) is deemed valid.
3. Fit the model (3) to *t*_*p*_ and *x* again but without reversing the censoring indicators. Under the null hypothesis *H*_0_ and with (9), the distribution of the Wald test statistic for ${\hat{\theta }}_{p}^{*}$ can be approximated by the standard normal distribution *N*(0,1).
4. Calculate the *p*‐value. Reject *H*_0_, if the *p*‐value is less than 0.05.

Note that even if *H*_0_ is not rejected, the pairwise method is still recommended because the unadjusted estimate ${\hat{\theta }}_{s}^{*}$ may still incorporate bias because of hidden balanced covariates and censoring, as illustrated in Figure 3(a) and (b).

## 5 Pairwise Cox likelihood

For the hazards (1) and (2), we first write the baseline hazard ratio as follows:

$$\frac{h_{0s}(t)}{h_{0p}(t)}=\exp\{\alpha (t)\}.$$

When evaluating effectiveness of treatments for many common chronic conditions such as cardiovascular disease, it is likely that *h*_0*s*_(*t*) > *h*_0*p*_(*t*) and so exp{*α*(*t*)}>1 for all $T_{\mathrm{pi}}=T_{\mathrm{si}}=t\geq 0$.

If we assume the baseline hazards *h*_0*s*_(*t*) and *h*_0*p*_(*t*) are proportional over time (analogous to the familiar Cox proportional hazards assumption), the log baseline hazard ratio is then equal to a constant *α*(*t*) = *α*. The parameter *α* can be regarded as a period effect. The hazards (1) and (2) now become

(10) $$h_{\mathrm{pi}}(t)=I(t\leq T_{\mathrm{pi}})h_{0p}(t)\exp(\beta C_{i})\;\;,$$

(11) $$\begin{array}{cc} h_{\mathrm{si}}(t) & =I(t\leq T_{\mathrm{si}})h_{0p}(t)\exp(\theta X_{i}+\beta C_{i}+\alpha )\\ =I(t\leq T_{\mathrm{si}})h_{0\mathrm{pi}}(t)\exp(\theta X_{i}+\alpha ), \end{array}$$

where the hazards (10) and (11) now share the same term *h*_0*pi*_(*t*) = *h*_0*p*_(*t*) exp(*β* *C*_*i*_), which can be regarded as an individual‐specific baseline hazard. For *n* individuals, the full likelihood is as follows:

(12) $$L_{\mathrm{full}}=\prod_{i}^{n}{\left\{h_{0\mathrm{pi}}\left(T_{\mathrm{pi}}\right)\right\}}^{\Delta_{\mathrm{pi}}}e^{-H_{0\mathrm{pi}}(T_{\mathrm{pi}})}{\left\{h_{0\mathrm{pi}}\left(T_{\mathrm{si}}\right)\exp\left(\theta X_{i}+\alpha \right)\right\}}^{\Delta_{\mathrm{si}}}e^{-H_{0\mathrm{pi}}(T_{\mathrm{si}})e^{\theta X_{i}+\alpha }},$$

where $H_{0\mathrm{pi}}(t)=\int_{0}^{t}h_{0\mathrm{pi}}(u)\mathrm{du}$ is the cumulative hazard function. It is clear that we cannot obtain a consistent estimate of *θ* using (12), because the number of nuisance parameters *h*_0*p*1_(*t*),…,*h*_0*pn*_(*t*) increases with the sample size *n*. We face the infinitely many nuisance parameters problem. The nuisance parameter *h*_0*pi*_(*t*) needs to be removed.

Suppose that *θ* and *α* are given, then as shown in Appendix A, the maximum likelihood estimators (MLEs) of *h*_0*pi*_(*t*) and *H*_0*pi*_(*t*) obtained from *L*_*full*_ are as follows:

(13) $$h_{0\mathrm{pi}}(T_{\mathrm{pi}})=\frac{\Delta_{\mathrm{pi}}}{1+P_{i}e^{\theta X_{i}+\alpha }},\;H_{0\mathrm{pi}}(T_{\mathrm{pi}})=\frac{\Delta_{\mathrm{pi}}}{1+P_{i}e^{\theta X_{i}+\alpha }}+\frac{S_{i}\Delta_{\mathrm{si}}}{S_{i}+e^{\theta X_{i}+\alpha }},$$

(14) $$h_{0\mathrm{pi}}(T_{\mathrm{si}})=\frac{\Delta_{\mathrm{si}}}{S_{i}+e^{\theta X_{i}+\alpha }},\;H_{0\mathrm{pi}}(T_{\mathrm{si}})=\frac{\Delta_{\mathrm{si}}}{S_{i}+e^{\theta X_{i}+\alpha }}+\frac{P_{i}\Delta_{\mathrm{pi}}}{1+P_{i}e^{\theta X_{i}+\alpha }},$$

where *P*_*i*_=*I*(*T*_*pi*_≤*T*_*si*_) and *S*_*i*_=*I*(*T*_*si*_≤*T*_*pi*_).

Plug the results (13) and (14) back into (12), and we get the pairwise Cox likelihood:

(15) $$L(\theta ,\alpha )=\prod_{i}^{n}{\left(\frac{1}{1+P_{i}e^{\theta X_{i}+\alpha }}\right)}^{\Delta_{\mathrm{pi}}}{\left(\frac{e^{\theta X_{i}+\alpha }}{e^{\theta X_{i}+\alpha }+S_{i}}\right)}^{\Delta_{\mathrm{si}}}.$$

The likelihood (15) is now free of the unknown term *h*_0*pi*_(*t*) = *h*_0*p*_(*t*)*e* *x* *p*(*β* *C*_*i*_).

As shown in web Appendix A, if we present the hazard models (10)–(11) and the likelihood (15) in the mathematical framework of counting processes using the same techniques in the proofs of Gross and Huber 17, it can be shown that the estimates $\hat{\theta }$ and $\hat{\alpha }$ from (15) are consistent and asymptotically normal. The covariance matrix can be estimated by the following:

$${\left[\overset{n}{\underset{i=1}{\sum }}e^{\hat{\theta }X_{i}+\hat{\alpha }}\left\{\frac{\Delta_{\mathrm{si}}S_{i}}{{\left(e^{\hat{\theta }X_{i}+\hat{\alpha }}+S_{i}\right)}^{2}}+\frac{\Delta_{\mathrm{pi}}P_{i}}{{\left(1+P_{i}e^{\hat{\theta }X_{i}+\hat{\alpha }}\right)}^{2}}\right\}\left(\begin{array}{cc} X_{i}X_{i}^{\mathrm{tr}} & X_{i}\\ X_{i}^{\mathrm{tr}} & 1 \end{array}\right)\right]}^{-1},$$

and the confidence interval can be constructed based on a normal approximation.

We can also show the consistency of the PERR‐ALT adjustment in Yu *et al*. 6. For the exposed group (*X*_*i*_=1), the hazards are as follows:

$$\begin{array}{cc} h_{\mathrm{pi}}(t) & =I(t\leq T_{\mathrm{pi}})h_{p0}(t)\exp(\beta C_{i})\\ h_{\mathrm{si}}(t) & =I(t\leq T_{\mathrm{si}})h_{p0}(t)\exp(\theta +\beta C_{i}+\alpha ). \end{array}$$

The pairwise likelihood is then

$$L(\theta +\alpha )=\underset{i}{\prod }{\left(\frac{1}{1+P_{i}e^{\theta +\alpha }}\right)}^{\Delta_{\mathrm{pi}}}{\left(\frac{e^{\theta +\alpha }}{e^{\theta +\alpha }+S_{i}}\right)}^{\Delta_{\mathrm{si}}}.$$

Because $\left(\hat{\theta },\hat{\alpha }\right)$ are consistent for (*θ*,*α*), the HR estimate for the exposed group (as denoted by ${\hat{\mathrm{HR}}}_{E}$ in Yu *et al*. 6) ${\hat{\mathrm{HR}}}_{E}=\exp(\hat{\theta +\alpha })$ is consistent for exp(*θ* + *α*).

For the unexposed group (*x*_*i*_=0), the hazards are the following:

$$\begin{array}{cc} h_{\mathrm{pi}}(t) & =I(t\leq T_{\mathrm{pi}})h_{p0}(t)\exp(\beta C_{i})\\ h_{\mathrm{si}}(t) & =I(t\leq T_{\mathrm{si}})h_{p0}(t)\exp(\beta C_{i}+\alpha ), \end{array}$$

and the paired likelihood is as follows:

$$L(\alpha )=\underset{i}{\prod }{\left(\frac{1}{1+p_{i}e^{\alpha }}\right)}^{\delta_{\mathrm{pi}}}{\left(\frac{e^{\alpha }}{e^{\alpha }+s_{i}}\right)}^{\delta_{\mathrm{si}}}.$$

Similarly, it can be shown that that the HR estimate for the unexposed group (as denoted by ${\hat{\mathrm{HR}}}_{\mathrm{uE}}$ in Yu *et al*. 6) ${\hat{\mathrm{HR}}}_{\mathrm{uE}}=\exp(\hat{\alpha })$ is consistent for the baseline hazard ratio exp(*α*).

Therefore, the PERR‐ALT estimate ${\hat{\mathrm{HR}}}_{\text{PERR‐ALT}}={\hat{\mathrm{HR}}}_{E}/{\hat{\mathrm{HR}}}_{\mathrm{uE}}$ is consistent for the true treatment effect exp(*θ*).

Note that the proportional baseline hazards assumption, log{*h*_0*s*_(*t*)/*h*_0*p*_(*t*)}=*α*(*t*) = *α*, is not compulsory for the pairwise Cox method. The pairwise Cox likelihood is flexible. We can, for example, assume the log‐baseline hazard ratio is linearly associated with time and thus specify *α*(*t*) = *α*_0_+*α*_1_ *t*. The pairwise likelihood is then as follows:

$$L(\theta ,\alpha_{0},\alpha_{1})=\overset{n}{\underset{i}{\prod }}{\left(\frac{1}{1+p_{i}e^{\theta x_{i}+\alpha_{0}+\alpha_{1}t_{\mathrm{pi}}}}\right)}^{\delta_{\mathrm{pi}}}{\left(\frac{e^{\theta x_{i}+\alpha_{0}+\alpha_{1}t_{\mathrm{si}}}}{e^{\theta x_{i}+\alpha_{0}+\alpha_{1}t_{\mathrm{si}}}+s_{i}}\right)}^{\delta_{\mathrm{si}}}.$$

For the example of diabetic retinopathy, the likelihood function is as follows:

(16) $$\prod_{i}^{n}{\left(\frac{e^{\theta_{p}X_{\mathrm{ip}}}}{e^{\theta_{p}X_{\mathrm{ip}}}+P_{i}e^{\theta_{s}X_{\mathrm{is}}+\alpha }}\right)}^{\Delta_{\mathrm{pi}}}{\left(\frac{e^{\theta_{s}X_{\mathrm{is}}+\alpha }}{e^{\theta_{s}X_{\mathrm{is}}+\alpha }+S_{i}e^{\theta_{p}X_{\mathrm{ip}}}}\right)}^{\Delta_{\mathrm{si}}}.$$

Because there are no biological differences between the left and right eyes, we can assume a common baseline hazard (*h*_0*p*_(*t*) = *h*_0*s*_(*t*)) and a common treatment effect on both eyes (*θ*_*p*_=*θ*_*s*_=*θ*). Therefore, *α* = log(*h*_0*s*_(*t*)/*h*_0*p*_) = 0, and the likelihood becomes

(17) $$\prod_{i}^{n}{\left(\frac{1}{1+P_{i}e^{\theta X_{i}}}\right)}^{\Delta_{\mathrm{pi}}}{\left(\frac{e^{\theta X_{i}}}{e^{\theta X_{i}}+S_{i}}\right)}^{\Delta_{\mathrm{si}}},$$

where *X*_*i*_=*X*_*is*_−*X*_*ip*_. We note that only patients receiving treatment on one of the eyes (i.e., *X*_*i*_=1 or −1) will contribute information to the likelihood (17).

### 5.1 Simulations

**Figure 4:**
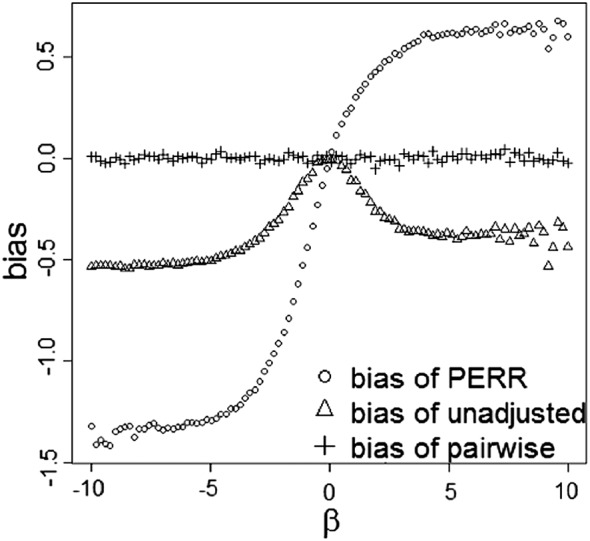
Simulation study of the bias of the unadjusted, prior event rate ratio (PERR) and pairwise methods in the case of a time‐varying baseline hazard. Graphs are based on simulated dataset of *n* = 100,000 paired event times. A Weibull baseline hazard *h*_0*p*_=2*t* was used. The main effect of treatment was *θ* = 1, and the period effect was *α* =− 1.

Figure 4 compares the biases of the unadjusted, PERR, and pairwise methods in the case of a time‐varying baseline hazard. We generated the prior and study event times from a Weibull model with *h*_0*p*_(*t*) = 2*t*,*θ* = 1,*α* =− 1,*β*∈(−10,10),*X* ∼ *B*(1,0.5),*C* ∼ *B*(1,0.3 + 0.4*X*), and both the prior and study data were 50*%* censored. The simulation results are similar to those in Figure 3(c) and show that the pairwise method is consistent.

Table 1 gives some examples of the variances for the unadjusted, PERR, and pairwise estimates under different censoring proportions for the prior and study periods. We generated *t*_*p*_ and *t*_*s*_ from (1) and (2), respectively, with *h*_0*p*_(*t*) = *θ* = *β* = 1, *h*_0*s*_(*t*)= exp(−0.5), *X* ∼ *B* *i* *n*(1,0.5), and *C*|*X* ∼ *B* *i* *n*(1,0.3 + 0.4*X*). We set censoring proportions for the prior and study periods as (10*%*,50*%*,90*%*). The average variance for each of the three methods and each value of the censoring proportion were calculated over 1000 simulation replications. For each replication, we set the number of events as 200 and the sample size as 200/{1− min(*C* *P*_*p*_,*C* *P*_*s*_)}, where *C* *P*_*p*_ and *C* *P*_*s*_ are the censoring proportions for prior and study periods, respectively. Table 1 shows that generally, the variances of the PERR and pairwise estimates are larger than those for the unadjusted estimates. The square root of the ratio between average variances shows how much the confidence interval of the pairwise method will be wider than that of the unadjusted method. It can been seen that the square root of variance ratio will generally increase with *C* *P*_*s*_−*C* *P*_*p*_.

**Table 1:** Examples of the variances for the unadjusted, PERR, and pairwise estimates under different censoring proportions in the prior and study periods.

Proportion of censoring in prior period	10*%*	10*%*	10*%*	50*%*	50*%*	50*%*	90*%*	90*%*	90*%*
Proportion of censoring in study period	10*%*	50*%*	90*%*	10*%*	50*%*	90*%*	10*%*	50*%*	90*%*
Average variance for unadjusted estimate	0.03	0.06	0.53	0.03	0.04	0.24	0.04	0.03	0.02
Average variance for PERR estimate	0.05	0.08	0.59	0.07	0.05	0.23	0.58	0.17	0.05
Average variance for pairwise estimate	0.08	0.14	0.91	0.13	0.07	0.41	0.61	0.38	0.08
Square root of the ratio between average variances for unadjusted and pairwise estimates	1.53	1.54	1.31	2.13	1.44	1.30	4.16	3.49	1.74

PERR, prior event rate ratio.

### 5.2 Treatment of ties and left truncation

In the special case where the prior and study times are tied $\left(\text{ie}t_{\mathrm{pi}}^{*}=t_{\mathrm{si}}^{*}\right)$, we assume the equality occurs because the measurement of event times is not accurate enough. The exact likelihood of the tied pair is hence the sum of the likelihoods in (13) if $t_{\mathrm{pi}}^{*}>t_{\mathrm{si}}^{*}$ and if $t_{\mathrm{pi}}^{*}<t_{\mathrm{si}}^{*}$. The sum is as follows:

$$\text{Exact likelihood}=L_{i}{\left(\theta ,\alpha \right)}_{\text{if}t_{\mathrm{pi}}^{*}>t_{\mathrm{si}}^{*}}+L_{i}{\left(\theta ,\alpha \right)}_{\text{if}t_{\mathrm{pi}}^{*}<t_{\mathrm{si}}^{*}}=\frac{1}{1+e^{\theta X_{i}+\alpha }}+\frac{e^{\theta X_{i}+\alpha }}{e^{\theta X_{i}+\alpha }+1}=1.$$

Therefore, the tied pairs make no contribution to the full likelihood and should be removed before using the pairwise Cox method. If the tied pairs are not removed, the partial likelihood in (15) will be the following:

(18) $$\frac{e^{\theta X_{i}+\alpha }}{{\left(1+e^{\theta X_{i}+\alpha }\right)}^{2}}\neq 1,$$

which is not identical to the exact likelihood, and thus, estimates of *θ* and *α* will be biased

Another issue that will be relevant in some studies is that of left truncation. For example, this can arise when the time at which a patient first becomes at risk of a prior event is unknown. For left truncation, there are two options. One is to redefine the events with different starting points to avoid the problem. For example, it may be reasonable to define the prior origin as the date of the patient's first medical record after reaching a particular age (or the date of diagnosis), even though the patient may have been at risk before that time. However, this option is not always feasible and may change the interpretation of parameters, potentially leading to violation of the assumptions of the hazard models. The other option is to impose a distributional assumption on the time of left truncation 12. The difficulty is that it is necessary to have reliable information on how to specify the distribution. More details can be found in section 4.5 of the book by Cook and Lawless 12.

### 5.3 Applications in crossover trials

As Figure 3(a) and (b) shows, the unadjusted Cox model will give biased estimates of treatment effects, even in randomized trials, if needed covariates are omitted. As it is never possible to capture all component causes 18, this potential for bias is likely to be present to some degree in all randomized controlled trials. In particular, it can have practical consequences for effectiveness estimates in smaller trials.

The pairwise Cox method can be applied with the crossover trial design to remove the bias from hidden covariates. As we have shown, even if there are infinitely many background covariates and it is impossible to measure all of them, as long as their values and effects are constant in (1) and (2), the relevant terms will only rescale the individual‐specific baseline hazard *h*_0*pi*_(*t*) and will be eliminated by the pairwise partial likelihood (15). However, as shown in Table 1, there is a trade‐off between bias and variance when using the pairwise method with crossover trials.

## 6 Limitations of prior event rate ratio adjustment

As with other methods for addressing hidden confounding, the validity of PERR adjustment based on the pairwise Cox likelihood relies on assumptions about the underlying causal model linking treatment (exposure) to outcomes. To assist applied researchers in deciding when the PERR method is likely to be of use in practice, we now review the key assumptions of the method and consider robustness of the method to violations of these assumptions:

Assumptions 1 Models (1) and (2) are correctly specified.

**Figure 5:**
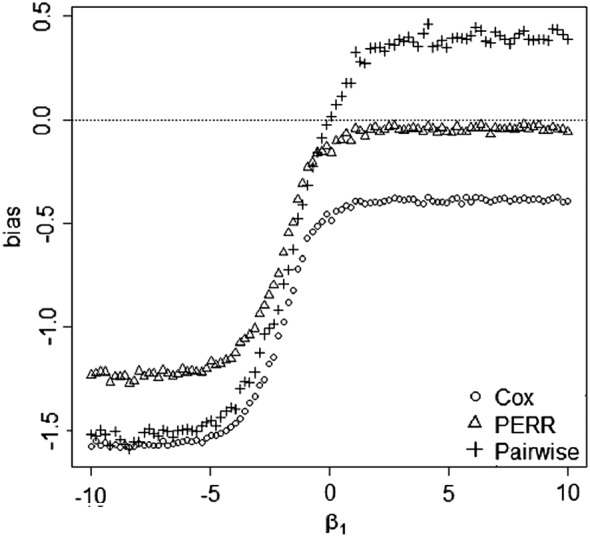
Simulation study of the bias of the pairwise Cox likelihood method and the original prior event rate ratio (PERR) adjustment method in the presence of an interaction between hidden confounder and treatment. Graphs are based on simulated dataset of *n* = 100,000 paired event times. An exponential baseline hazard was used with a rate parameter of 1. The main effect of treatment was *θ* = 1, and the period effect was *α* = 1. *β*_1_ denotes the size of the interaction effect.

If the models are misspecified, for example, if there is an interaction between treatment and the unmeasured confounder(s) or the value and effect of the unmeasured confounder are time‐varying, the pairwise method will be biased. Uddin *et al*. 19 studied the performance of the PERR estimator when the effect of the unmeasured confounder varies between periods and showed that the PERR method gives biased estimates of the rate ratio, but the bias is generally smaller than for conventional analyses. Figure 5 shows an example of the biases for the pairwise Cox likelihood method, the original PERR adjustment method, and the standard Cox regression model for different degrees of confounder‐treatment interaction. The paired event times *t*_*p*_ and *t*_*s*_ were generated from (1) and *h*_0*p*_(*t*) exp(*θ* *X*_*i*_+*β* *C*_*i*_+*α* + *β*_1_ *X*_*i*_ *C*_*i*_) with *h*_0*p*_(*t*) = *θ* = *α* = *β* = 1, *X* ∼ *B*(1,0.5), and *C*|*X* ∼ *B*(1,0.3 + 0.4*X*). The sample size was 100,000, and there was no censorship. The bias was estimated for different values of the interaction effect *β*_1_.

It can be seen that when the true hazard model contains an interaction, the bias curve for the pairwise Cox method has a similar sigmoid shape to the curves for the Cox model $\left(\text{i.e.}h_{0s}^{*}(t)e^{\theta_{s}^{*}X}\right)$ and the original PERR method. When the magnitude of the interaction effect is less than that of the main effect of treatment, that is, |*β*_1_|<1, the pairwise method reduces the bias compared with the Cox model. However, the figure illustrates that there may be regions, corresponding to more extreme degrees of model misspecification, where the Cox model performs no worse than or better than PERR adjustment.

Assumptions 2 Prior event occurrence should not influence likelihood of future treatment/exposure.

**Figure 6:**
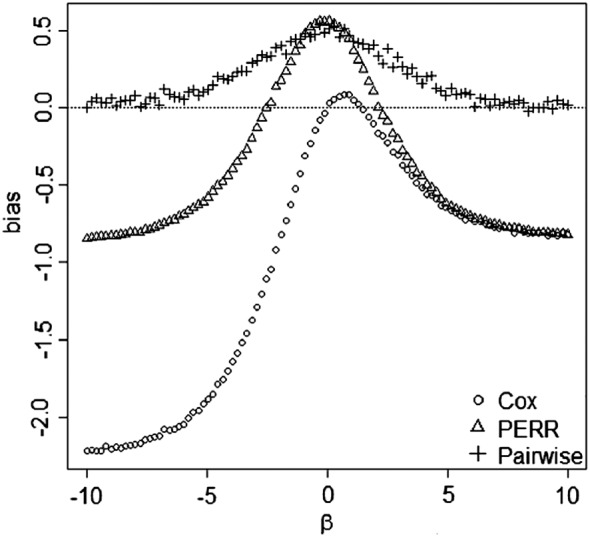
Simulation study of the bias of the pairwise Cox likelihood method and the original prior event rate ratio (PERR) adjustment method when prior events influence the likelihood of future receipt of treatment. Graphs are based on simulated dataset of *n* = 100,000 paired times *t*_*p*_ and *t*_*s*_ generated from (1) and (2). An exponential baseline hazard was used with a rate parameter of 1. The censoring mechanism was uniform, and both the prior and study event data were 50*%* censored. The main effect of treatment was *θ* = 1, and the period effect was *α* = 1. *C* and *X*_*i*_ were generated from ((19)) so that the treatment variable was associated with the unmeasured confounder and the prior censoring indicator *δ*_*pi*_.

Gallagher *et al*. 20 and Uddin *et al*. 19 showed that the original PERR is biased when the likelihood of treatment is affected by the prior event occurrence. We assessed the performance of the pairwise method under this problem and found that it also gave biased estimates of treatment effects. An example of this bias is illustrated in Figure 6. The paired times *t*_*p*_ and *t*_*s*_ were generated from (1) and (2) with *h*_0*p*_(*t*) = *θ* = *α* = *β* = 1. The sample size was 100,000. The censoring mechanism was uniform, and both the prior and study event data were 50*%* censored. We generated

(19) $$C∼B(1,0.5)\;\text{and}\;X_{i}∼B\left(1,\text{expit}\left(C_{i}-\Delta_{\mathrm{pi}}\right)\right)$$

so that the treatment variable was associated with the unmeasured confounder and the prior censoring indicator *δ*_*pi*_. Understanding the mechanism underlying the bias and developing possible solutions is an interesting open question in its own right.

Remarks on the problem of differential case fatality: Gallagher *et al*. 20 also showed that the original PERR method is biased in the case of differential case fatality (i.e., where high‐risk patients are more likely to die before reaching exposure).

We conducted a simulation study to further explore the performance of the PERR and pairwise methods in the presence of differential case fatality (web Appendix B). The underlying bias of the PERR estimator (as shown in Section 3) was shown to change when case fatality was differential (both increases or decreases in bias were possible). However, we found that the pairwise method was unbiased in this situation. The reason is that the pairwise method compares the outcomes of the same patient before and after study and thus differential case fatality will not introduce bias. In contrast, for the PERR method, the distribution of the unmeasured confounder is changed when case fatality is differential. For example, suppose *C* ∼ *Bin*(1,0.5) at the beginning of the prior period and the groups with *c* = 0(low risk) and *c* = 1 (high risk) have differential case fatality of 10*%* and 50*%*, respectively, then the distribution of *C* for the subjects in the PERR analysis becomes $\text{Bin}\left(1,\frac{0.5\times 0.5}{0.5\times 0.9+0.5\times 0.5}\right)$. As a result, differential case fatality changes the distribution of the unmeasured confounder and thus changes the bias of PERR. In fact, the bias of PERR in this case is the same as the bias in the absence of differential case fatality with $C∼\text{Bin}\left(1,\frac{0.5\times 0.5}{0.5\times 0.9+0.5\times 0.5}\right)$. This change in the bias due to differential case fatality corresponds to the difference between the PERR biases for the two unmeasured confounding distributions (web Appendix B). We note that in the extreme case that all the subjects with *c* = 1 die before exposure and all the subjects with *c* = 0 survive to exposure, the PERR method (as well as the unadjusted Cox model) will be unbiased because all the subjects in the analysis have the same value of *c* = 0.

## 7 More general likelihoods

### 7.1 Time‐varying covariates

The pairwise likelihood (15) can be extended to allow flexible modeling in more general situations. For example, in practice, we may have some covariates measured in the prior period, and their values and effects might change over time. Adopting a commonly used model for time‐varying covariates, the hazards can be written as follows:

(20) $$h_{\mathrm{pi}}(t)=I(t\leq T_{\mathrm{pi}})h_{0p}(t)\exp\left\{\theta (t)x_{i}(t)+\beta c_{i}\right\},$$

(21) $$h_{\mathrm{si}}(t)=I(t\leq T_{\mathrm{si}})h_{0p}(t)\exp\left\{\theta (t+\tau_{\mathrm{pi}})x_{i}(t+\tau_{\mathrm{pi}})+\beta c_{i}+\alpha \right\},$$

where *τ*_*pi*_ is the time span between the starting points of the prior and study periods for the *i*th individual. The models (20) and (21) assume that the hazards at time *t*_*pi*_=*t*_*si*_=*t* are only affected by the current value of the covariate and its effect at the time *t*_*pi*_=*t*_*si*_=*t*. Unbiased estimates of the model parameters can be obtained from the following:

(22) $$L(\theta (t),\alpha )=\prod_{i}^{n}{\left\{\frac{e^{\theta (t_{\mathrm{pi}})x_{i}(t_{\mathrm{pi}})}}{e^{\theta (t_{\mathrm{pi}})x_{i}(t_{\mathrm{pi}})}+p_{i}e^{\theta (t_{\mathrm{pi}}+\tau_{\mathrm{pi}})x_{i}(t_{\mathrm{pi}}+\tau_{\mathrm{pi}})+\alpha }}\right\}}^{\delta_{\mathrm{pi}}}{\left\{\frac{e^{\theta (t_{\mathrm{si}}+\tau_{\mathrm{pi}})x_{i}(t_{\mathrm{si}}+\tau_{\mathrm{pi}})+\alpha }}{e^{\theta (t_{\mathrm{si}}+\tau_{\mathrm{pi}})x_{i}(t_{\mathrm{si}}+\tau_{\mathrm{pi}})+\alpha }+s_{i}e^{\theta (t_{\mathrm{si}})x_{i}(t_{\mathrm{si}})}}\right\}}^{\delta_{\mathrm{si}}}.$$

The simple hazard models (10) and (11) and the likelihood (15) can be regarded as the special cases of (20), (21), and (22), respectively, when

$$\theta (t)x_{i}(t)=\left\{\begin{array}{ll} 0, & \text{for the hazard of prior event}\\ \theta x_{i}, & \text{for the hazard of study event} \end{array}\right..$$

#### 7.1.1 An example with period‐specific treatment effects

Suppose the time‐varying covariates and effects take the values

$$\theta (t)X_{i}(t)=\left\{\begin{array}{ll} \theta_{p}x_{\mathrm{pi}}, & \text{for the hazard of prior event}\\ \theta_{s}x_{\mathrm{si}}, & \text{for the hazard of study event} \end{array}\right..$$

Such a model could be used for considering the effects of treatments, such as statins, for which effects on outcomes or side effects may not become apparent until after an initial period on treatment. The corresponding hazards are as follows:

(23) $$h_{\mathrm{pi}}(t)=I\left(t\leq T_{\mathrm{pi}}\right)h_{0p}(t)\exp\left(\theta_{p}x_{\mathrm{pi}}+\beta c_{i}\right),$$

(24) $$h_{\mathrm{si}}(t)=I\left(t\leq T_{\mathrm{si}}\right)h_{0p}(t)\exp\left(\theta_{s}x_{\mathrm{si}}+\beta c_{i}+\alpha \right),$$

where *θ*_*s*_ is the parameter of interest. The likelihood is then

(25) $$L(\theta_{s},\theta_{p},\alpha )=\prod_{i}^{n}{\left\{\frac{e^{\theta_{p}x_{\mathrm{pi}}}}{e^{\theta_{p}x_{\mathrm{pi}}}+p_{i}e^{\theta_{s}x_{\mathrm{si}}+\alpha }}\right\}}^{\delta_{\mathrm{pi}}}{\left\{\frac{e^{\theta_{s}x_{\mathrm{si}}+\alpha }}{e^{\theta_{s}x_{\mathrm{si}}+\alpha }+s_{i}e^{\theta_{p}x_{\mathrm{pi}}}}\right\}}^{\delta_{\mathrm{si}}}.$$

A simulation study was conducted to compare the performance of the likelihood (25) with the unadjusted hazard model $h(t_{\mathrm{si}})=h_{0s}^{*}(t_{\mathrm{si}})\exp\left(\theta_{s}^{*}x_{\mathrm{si}}\right)$. We generated 100,000 paired event times (*t*_*pi*_,*t*_*si*_) from ((23)) and ((24)) with *h*_0*p*_(*t*) = *θ*_*s*_=1, *θ*_*p*_=*α* =− 1, *β* = (−10,10), *X*_*p*_∼*Bin*(1,0.5), *X*_*s*_∼*Bin*(1,0.7 − 0.4*X*_*p*_), and *C* ∼ *Bin*(1,0.3 + 0.4*X*_*s*_). The censoring mechanism was uniform, and both the prior and study event data were 50*%* censored. The biases of the pairwise method were calculated by ${\hat{\theta }}_{s}-\theta_{s}$, where ${\hat{\theta }}_{s}$ was obtained from (25). The bias of the unadjusted hazard ratio was ${\hat{\theta }}_{s}^{*}-\theta_{s}$.

**Figure 7:**
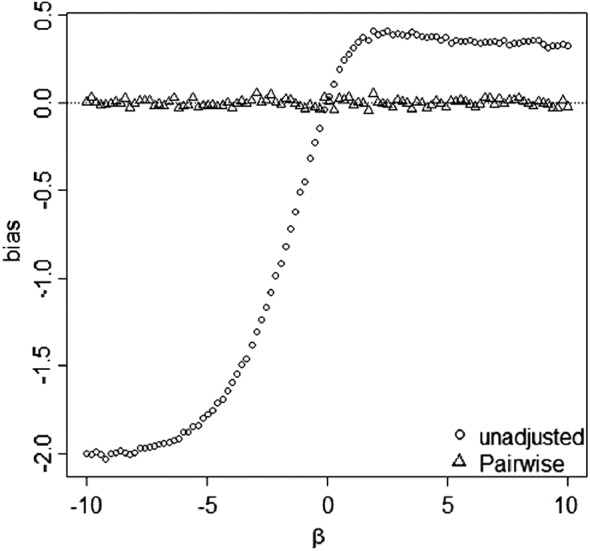
Simulation study of the pairwise method (25) and the unadjusted hazard model $h(t_{\mathrm{si}})=h_{0s}^{*}(t_{\mathrm{si}})\exp\left(\theta_{s}^{*}x_{\mathrm{si}}\right)$ in the presence of time‐varying covariates and effects. Graphs are based on a simulated dataset with *n* = 100,000 and log‐hazard ratio of treatment *θ*_*s*_=1 and *θ*_*p*_=−1. An exponential baseline hazard was used with a rate parameter of *h*_0_(*t*) = 1. The censoring rate is 50*%* for both prior and study periods.

The results presented in Figure 7 show that the pairwise method eliminates the bias in the unadjusted model. The PERR method was not considered here because it is not applicable in this case.

### 7.2 More than two periods

The likelihood can also be extended to the case of more than two periods. Suppose the hazard of the *i*th individual of experiencing the *k*th event is as follows:

$$h_{\mathrm{ik}}(t)=I\left(t\leq T_{\mathrm{ik}}\right)h_{0}(t)\exp\left\{\beta c_{i}+\theta \left(t+\tau_{\mathrm{ik}}\right)x_{i}\left(t+\tau_{\mathrm{ik}}\right)+\alpha_{k}\right\},\;k=1,2,\ldots ,$$

where *h*_0_(*t*) is the baseline hazard of the first event, *τ*_*ik*_ is the time span between the starting points of the first period and the *k*th period, and *α*_*k*_ is the log ratio of baseline hazards between the *k*th and the first events with *α*_1_=0.

For a sample of *n* individuals and the *i*th individual with *m*_*i*_ events, the partial likelihood is as follows:

(26) $$L=\prod_{i=1}^{n}\prod_{k=1}^{m_{i}}{\left(\frac{e^{\theta (t_{\mathrm{ik}}+\tau_{\mathrm{ik}})x_{i}(t_{\mathrm{ik}}+\tau_{\mathrm{ik}})+\alpha_{k}}}{\sum_{j=1}^{m_{i}}I(t_{\mathrm{ik}}\leq t_{\mathrm{ij}})e^{\theta (t_{\mathrm{ik}}+\tau_{\mathrm{ij}})x_{i}(t_{\mathrm{ik}}+\tau_{\mathrm{ij}})+\alpha_{j}}}\right)}^{\delta_{\mathrm{ik}}}.$$

In the case of ties, ((26)) is a Breslow approximation to the exact likelihood, which should work well if the ratio between the number of ties and *m*_*i*_ is small. However, if this condition is not satisfied, for example, *m*_*i*_=2, the approximation can be poor as shown in ((18)). In this situation, the exact likelihood or Efrons approximation would be suggested.

## 8 Application: comparative effectiveness of enzyme replacement therapies for patients with Fabry disease

### 8.1 Background and model specification

We consider application of the pairwise Cox approach to data from a longitudinal cohort study of enzyme replacement therapy (ERT) for Fabrys disease. Fabry disease is a rare, inherited metabolic disorder resulting from absolute or partial deficiency of the enzyme *α*‐galactosidase A and the progressive accumulation of undigested macromolecules in cells throughout the body, particularly in the heart, kidneys, and nerve tissue 21. There are two forms of enzyme replacement products approved for use in the USA and Europe: agalsidase alfa and agalsidase beta. Studying the comparative efficacy and safety of these two therapies is of current clinical relevance as there is a tendency for the drug of choice to vary between treatment centers, often for historical reasons 21. The rarity and severity of this condition have resulted in a lack of adequately powered randomized trials for making such comparisons, and so researchers have instead had to rely on observational studies, with the attendant risk of bias from hidden confounding. We fitted the pairwise Cox model to estimate comparative effectiveness of the two forms of ERT using data from the National Collaborative Study for Lysosomal Storage Disorders, a longitudinal cohort study collecting both prospective and historical data 22. Here, the focus is on illustrating usage of the methods rather than drawing firm conclusions about the treatments involved, and so we refer to the treatments simply as therapy A and therapy B. Our analyses necessarily involve simplification of the issues that would be involved in substantive analyses of the source data, and so the results we present should not be used to draw inferences for clinical practice.

Overall, a total of 211 adults with Fabry disease were being treated with ERT on recruitment to the National Collaborative Study for Lysosomal Storage Disorders study. For this illustrative example, the outcome of interest was estimated glomerular filtration rate (eGFR), a measure of kidney function, and we analyzed data for the 45 patients with at least two measures of eGFR, both before and after starting therapy. The data for the 45 patients are provided in web Appendix C and is available to download as a CSV file. Of these patients, 26 received therapy A, and 19 were on therapy B. Two potential confounding variables, age and gender, were available for this analysis. The patients on therapy A were younger and more likely to be female (mean age of starting therapy =41.0 years; 35*%* female) than patients on therapy B (mean age of starting therapy =48.1 years; 5*%* female). We were unable to consider other potential confounding variables, such as genotype and disease severity, because of a lack of consistency in the way that they were assessed and recorded. The aim of the pairwise Cox analysis was to account for these unmeasured differences. Given the modest sample size, the analysis can be seen as a pilot study to inform the design of a more extensive follow‐up study.

To facilitate application of the pairwise Cox method, the multiple renal function measures were converted into time‐to‐event data by defining the prior and study events as the first times when eGFRs were greater than those in the first visits before treatment and the visits at or following treatment, respectively (in the absence of measurement error, increases in eGFR correspond to improvements in kidney function). According to these definitions of the prior and study events, the start of the prior period was the first visit recorded in the database before treatment, and the corresponding start of the study period was the visit at or following the time of initiating ERT therapy. The observed prior and study event times, *T*_*p*_ and *T*_*s*_, were measured from the start of the prior and study periods to the prior and study events (or to the times of receiving therapies and the end of study), respectively. The prior and study periods are terminated at *T*_*p*_ and *T*_*s*_, respectively. We note that defining the study origin as the visit at or following the point of starting ERT therapy was a consequence of the definition of a study event. This helped simplify the problem for the purposes of this illustrative example. In practice, it is inappropriate to set the time of receiving treatment as the study origin, if the time of receiving treatment is not the time when an individual starts to be under the risk of the defined study event.

Let *C*_*i*_ be an unmeasured confounder, *X*_*i*5_ be the indicator of gender (0= male; 1= female), *X*_*i*3_ and *X*_*i*4_ be the ages in years at the beginning of the prior and study periods, respectively, and *X*_*i*1_ and *X*_*i*2_ be the treatment indicators (no treatment: *X*_*i*1_=*X*_*i*2_=0; therapy A: *X*_*i*1_=1,*X*_*i*2_=0; therapy B: *X*_*i*1_=0,*X*_*i*2_=1). The hazard functions of the prior and study events for the *i*th patient are assumed to be as follows:

(27) $$h_{\mathrm{pi}}(t)=I\left(t\leq T_{\mathrm{pi}}\right)h_{0p}(t)e^{\beta C_{i}+\theta_{5}X_{i5}+\theta_{3}(X_{i3}+t)}\;\;,$$

(28) $$h_{\mathrm{si}}(t)=I\left(t\leq T_{\mathrm{si}}\right)h_{0s}(t)e^{\beta C_{i}+\theta_{5}X_{i5}+\theta_{3}(X_{i4}+t)+\theta_{1}X_{i1}+\theta_{2}X_{i2}},$$

(29) $$=I\left(t\leq T_{\mathrm{si}}\right)h_{0\mathrm{pi}}e^{\left(\theta_{1}-\theta_{2})X_{i1}+\alpha +\theta_{2}+\theta_{3}(X_{i4}-X_{i3}\right)},$$

where *β*, *θ*_5_,*θ*_3_,*θ*_1_, and *θ*_2_ are the coefficients and *α* = log{*h*_0*s*_(*t*)/*h*_0*p*_(*t*)}.

Note that in the hazard models (27)–(29), age is a time‐varying covariate measured in both the prior and study periods and taking the values *X*_*i*3_+*t* and *X*_*i*4_+*t* at the times of prior and study events, respectively. Because the prior and study events are of the same nature, that is, renal function increases, the coefficient *θ*_5_ (or *θ*_3_) is assumed to be the same in the hazards (27) and (28). Otherwise, we would need to specify different *θ*_5_ (or *θ*_3_), such as using *θ*_5*p*_ in (27) and *θ*_5*s*_ in (28). As all the patients received one of the two types of ERT therapy and no one was untreated, *X*_*i*2_=1 − *X*_*i*1_, and we are unable to estimate *θ*_1_ and *θ*_2_. But the difference between therapy A and therapy B, *θ*_1_−*θ*_2_, can be estimated.

### 8.2 Estimates under the pairwise, unadjusted Cox models and prior event rate ratio

The pairwise likelihood is as follows:

(30) $$L(\theta_{1}-\theta_{2},\alpha +\theta_{2},\theta_{3})=\prod_{i}^{n}{\left\{\frac{1}{1+P_{i}e^{\left(\theta_{1}-\theta_{2})X_{i1}+\alpha +\theta_{2}+\theta_{3}(X_{i4}-X_{i3}\right)}}\right\}}^{\Delta_{\mathrm{pi}}}{\left\{\frac{e^{\left(\theta_{1}-\theta_{2})X_{i1}+\alpha +\theta_{2}+\theta_{3}(X_{i4}-X_{i3}\right)}}{e^{\left(\theta_{1}-\theta_{2})X_{i1}+\alpha +\theta_{2}+\theta_{3}(X_{i4}-X_{i3}\right)}+S_{i}}\right\}}^{\Delta_{\mathrm{si}}}.$$

The likelihood of the Cox model fitted to the study data without adjustment for *C*_*i*_ is as follows:

(31) $$L(\theta_{5},\theta_{3},(\theta_{1}-\theta_{2}))=\prod_{i}^{n}{\left[\frac{e^{\theta_{5}X_{i5}+\theta_{3}(X_{i4}+T_{\mathrm{si}})+(\theta_{1}-\theta_{2})X_{i1}}}{\sum_{j:T_{\mathrm{sj}}\geq T_{\mathrm{si}}}e^{\theta_{5}X_{j5}+\theta_{3}(X_{j4}+T_{\mathrm{si}})+(\theta_{1}-\theta_{2})X_{j1}}}\right]}^{\Delta_{\mathrm{si}}}.$$

The likelihood of the Cox model fitted to the prior data without adjustment for *C*_*i*_ is as follows:

(32) $$L(\theta_{5},\theta_{3},(\theta_{1}-\theta_{2}))=\prod_{i}^{n}{\left[\frac{e^{\theta_{5}X_{i5}+\theta_{3}\left(X_{i3}+T_{\mathrm{pi}}\right)+(\theta_{1}-\theta_{2})X_{i1}}}{\sum_{j:T_{\mathrm{pj}}\geq T_{\mathrm{pi}}}e^{\theta_{5}X_{j5}+\theta_{3}\left(X_{j3}+T_{\mathrm{pi}}\right)+(\theta_{1}-\theta_{2})X_{j1}}}\right]}^{\Delta_{\mathrm{pi}}}.$$

The estimates of $e^{\theta_{1}-\theta_{2}}$ under the pairwise model (30) and the unadjusted Cox models (31) and (32) are presented in Table 2 with 95*%* confidence intervals obtained from the observed information. The R code for the pairwise model (30) is provided in Appendix B. The PERR estimate is 1.25/0.93 = 1.34, and the 95*%* confidence interval was obtained by the bootstrap method. The estimates for the pairwise, unadjusted and PERR methods are consistently above 1, but the differences are not significant at the 5*%* level. Use of the pairwise method has led to an increased point estimate of 1.55, suggesting there may be the potential to uncover genuine differences between therapies in a larger study.

**Table 2:** Estimates of the hazard ratio for the difference between ERT therapies using the pairwise Cox method, the unadjusted Cox model, and the PERR method, for the example data on patients with Fabry disease.

Model	*HR* $e^{\hat{\theta_{1}-\theta_{2}}}$	95*%* CI	*p*‐value
Pairwise (30)	1.55	(0.70,3.14)	0.28
Unadjust (31)	1.25	(0.59,2.66)	0.57
Unadjust prior (32)	0.93	(0.42,2.05)	0.86
PERR	1.34	(0.31,5.74)	0.69

PERR, prior event rate ratio.

### 8.3 Using the prior data to detect unmeasured confounding

To make use of the method in Section 4, we first test whether the assumptions (*X*_5_,*X*_3_)⊥*X*_1_ and $S_{p}^{+}(t|X_{1},X_{5},X_{3})=S_{p}^{+}(t|X_{5},X_{3})$ hold. We assessed these by fitting a logistic regression to *X*_1_ on *X*_3_ and *X*_5_, and a Cox model to *t*_*p*_ on *X*_1_,*X*_3_,*X*_5_ with the censoring indicator swapped. There was no significant evidence against either of these assumptions (the *p*‐values for likelihood ratio tests are 0.14 and 0.88, respectively ). Under these assumptions, the *p*‐value of the unadjusted Cox model (32) provides a test for hidden confounding. In this example, there was no significant evidence of hidden confounding (*p* = 0.86), perhaps due to lack of power to detect an effect in this small sample. However, as mentioned in Section 4, the unadjusted Cox model and the PERR will underestimate the treatment effect, even if the unmeasured covariate is not a confounder. The pairwise method is still recommended in the absence of significant evidence of hidden confounding.

## 9 Discussion

This paper is concerned with identifying and reducing the impact of hidden confounding in observational studies of treatment effectiveness. Building on the work of Tannen and colleagues, we set out a general framework for quasi‐experimental analysis using the PERR approach and derived a flexible pairwise Cox likelihood function that can be used to estimate unbiased treatment estimates, under appropriate assumptions. The pairwise likelihood was used to demonstrate the consistency of the simple and convenient PERR‐ALT estimator introduced by Yu *et al*. 6. We showed how to estimate standard errors and confidence intervals for treatment effect estimates based on the pairwise model and provided R code to illustrate how to implement the method. A simple test to detect unmeasured confounding was also developed based on data from a prior period.

Much of the previous work on PERR adjustment has focused on analysis of electronic medical record databases. While the growth of large‐scale data registries makes this a particularly important application, the approach also has potential value in population‐based research studies. This was illustrated through an example in which the pairwise method was applied to a longitudinal cohort study combining prospective data collection with retrospective use of routine health records.

Designing future quasi‐experimental studies that exploit the pairwise Cox method requires an understanding of the power of the method. We showed that the variance for the pairwise method is usually larger than that for a conventional Cox regression analysis. In the era of large electronic medical record databases, the additional variance is unlikely to be overly burdensome unless the focus is on narrow segments of the population.

One of the strengths of the pairwise Cox likelihood approach is its inherent flexibility. Like the conventional Cox model, the pairwise method can easily be extended to accommodate time‐varying treatment covariates and coefficients. Furthermore, the form of *α*(*t*), corresponding to the period effect, can be chosen by comparing models with different forms of *α*(*t*), making it possible to relax the proportional baseline hazards assumption. In addition, we leave the user free to define the prior and study events for different research problems, without the need for the prior and study periods to be successive.

Several limitations of the PERR approach need to be considered by investigators conducting observational research. Yu *et al*. 6 highlight the fundamental requirement for prior events. Consequently, in its current form, the pairwise Cox likelihood cannot be used for evaluating treatment effects on mortality or other terminal events. The method also relies on strong assumptions about model specification including the absence of time‐varying hidden confounders and confounder by treatment interaction. In practice, the method may be robust to realistic levels of departure from these assumptions 6, but as we have demonstrated in extreme cases, the method can be as biased, or even more biased, than conventional approaches.

An additional challenge to the validity of the method arises when treatment allocation is associated with prior events, such as if a patient is switched to a new treatment following failure of an existing treatment. Consistent with the findings of Gallagher *et al*. 20, our simulations highlighted that both pairwise and PERR methods are likely to be biased in this situation. These findings reinforce the need to consider the context of the problem carefully when assessing the suitability of the PERR approach. In its current form, the pairwise method may be most useful where the decision to start treatment is not likely to be related to the risk of future events, for example, in drug safety monitoring or evaluations of national vaccination programs.

A number of other biostatistical methods for tackling hidden confounding have been proposed. Common approaches that provide estimates of causal effects include propensity scoring combined with regression calibration 23, instrumental variables 24, and regression discontinuity designs 25. In practice, no one method is likely to be best in all problems, and it is essential for investigators to carefully assess the potential biases in each proposed study, where possible tailoring the methods or combination of methods to address these biases 2. Tannen*et al*. 5 provided preliminary evidence that the PERR approach can produce reliable results when replicating outcomes of cardiovascular trials, but further empirical studies are needed to establish the validity of the method for use in other clinical problems, and to determine the strengths and limitations of the PERR approach relative to other methods. Where possible, this should include proof of concept studies to replicate results of randomized trials as well as clinically informed simulation studies.

Like the instrumental variable method that requires a suitable instrument and propensity score calibration that needs a suitable validation study, the pairwise method can be only used when suitable data are available. For example, data on prior events will not be available in some problems, such as a primary prevention study of statin therapy for patients with no prior history of cardiovascular disease. However, the pairwise method is likely to become more accessible with the increasing availability of large electronic medical record databases, because such datasets can often provide the necessary volumes of longitudinal data (albeit of variable quality and completeness) before and after patients receive treatments.

In conclusion, the PERR and pairwise Cox methods offer a promising approach to addressing biases that can arise in observational studies because of lack of randomization and through further development could become a highly cost‐effective way of using established datasets to answer questions about treatment effectiveness in clinical practice. The flexibility of the pairwise Cox likelihood offers a basis for generalizing the method, but widespread adoption of the approach will require further progress in addressing the challenges of dealing with prior events that influence treatment, terminal events, and the presence of time‐varying confounding.

## Data Accessibility

Data pertaining to this manuscript is deposited in figshare at DOI: http://dx.doi.org/10.6084/m9.figshare.3470384

- Data for Web Appendix C.csv. This file contains raw data from a longitudinal cohort study of enzyme replacement therapy for patients with Fabry disease. The data are analysed in the example given in Section 8 of the published paper.

## Supporting information

Supporting info item

## Appendix A Estimate of $h_{0pi}(\cdot )$

### A.1

The log full likelihood is as follows:

$$l_{\mathrm{full}}=\overset{n}{\underset{i}{\sum }}\left[\Delta_{\mathrm{pi}}\log\left\{h_{0\mathrm{pi}}\left(T_{\mathrm{pi}}\right)\right\}-H_{0\mathrm{pi}}\left(T_{\mathrm{pi}}\right)+\Delta_{\mathrm{si}}\left[\log\left\{h_{0\mathrm{pi}}\left(T_{\mathrm{si}}\right)\right\}+\theta X_{i}+\alpha \right]-H_{0\mathrm{pi}}\left(T_{\mathrm{si}}\right)e^{\theta X_{i}+\alpha }\right].$$

Using the argument similar to Johansen 26, we assume the hazard is 0, *h*_0*p**i*_(*t*) = 0, except at event times and the cumulative hazard is the summation of the hazards, that is,

(A.1) $$h_{0\mathrm{pi}}\left(T_{\mathrm{pi}}\right)=\Delta_{\mathrm{pi}}h_{\mathrm{pi}}\;H_{0\mathrm{pi}}\left(T_{\mathrm{pi}}\right)=\Delta_{\mathrm{pi}}h_{\mathrm{pi}}+S_{i}\Delta_{\mathrm{si}}h_{\mathrm{si}},$$

(A.2) $$h_{0\mathrm{pi}}\left(T_{\mathrm{si}}\right)=\Delta_{\mathrm{si}}h_{\mathrm{si}}\;H_{0\mathrm{pi}}\left(T_{\mathrm{si}}\right)=\Delta_{\mathrm{si}}h_{\mathrm{si}}+P_{i}\Delta_{\mathrm{pi}}h_{\mathrm{pi}}.$$

Then *l*_*full*_ becomes

$$\begin{array}{cc} l_{\text{full}} & =\overset{n}{\underset{i}{\sum }}\left[\Delta_{\mathrm{pi}}\log\left(\Delta_{\mathrm{pi}}h_{\mathrm{pi}}\right)-\Delta_{\mathrm{pi}}h_{\mathrm{pi}}-S_{i}\Delta_{\mathrm{si}}h_{\mathrm{si}}+\Delta_{\mathrm{si}}\left\{\log\left(\Delta_{\mathrm{si}}h_{\mathrm{si}}\right)+\theta X_{i}+\alpha \right\}\right.\\ \;\;\left.-\left(\Delta_{\mathrm{si}}h_{\mathrm{si}}+P_{i}\Delta_{\mathrm{pi}}h_{\mathrm{pi}}\right)e^{\theta X_{i}+\alpha }\right]. \end{array}$$

Keeping *θ* and *α* fixed, the MLEs of *h*_*p**i*_ and *h*_*s**i*_ are the following:

$$\begin{array}{cc} \frac{\partial l_{\text{full}}}{\partial h_{\mathrm{pi}}} & =\frac{\Delta_{\mathrm{pi}}}{h_{\mathrm{pi}}}-\Delta_{\mathrm{pi}}-P_{i}\Delta_{\mathrm{pi}}e^{\theta X_{i}+\alpha }\;\Rightarrow \;h_{\mathrm{pi}}=\frac{1}{1+P_{i}e^{\theta X_{i}+\alpha }}\\ \frac{\partial l_{\text{full}}}{\partial h_{\mathrm{si}}} & =-S_{i}\Delta_{\mathrm{si}}+\frac{\Delta_{\mathrm{si}}}{h_{\mathrm{si}}}-\Delta_{\mathrm{si}}e^{\theta X_{i}+\alpha }\;\Rightarrow \;h_{\mathrm{si}}=\frac{1}{S_{i}+e^{\theta X_{i}+\alpha }}. \end{array}$$

These together with ((A.1)) and ((A.2)) lead to the results (13) and (14).

## Appendix B R code for the example

### B.1

The log‐likelihood function of (30) is as follows:
