# Supplementary material for: Prior event rate ratio adjustment for hidden confounding in observational studies of treatment effectiveness: a pairwise Cox likelihood approach
Source: Stat Med. 2016 Aug 1;35(28):5149–69. doi: 10.1002/sim.7051 (PMC5111612; doi:10.1002/sim.7051)
Supplement: Supplementary file 1 — Supporting info item [file SIM-35-5149-s001.zip › Data/SIM_7051_supp0001-Web Appendix.pdf]

Web Appendix for Prior Event Rate Ratio Adjustment for Hidden Confounding in Observational Studies of Treatment Effectiveness: A Pairwise Cox Likelihood Approach

Web Appendix A: Consistency and asymptotic normality

For matched pair experiments, Gross and Huber [1] considered the following counting process

$$N_{ij}(t) = \begin{cases} 1 & \text{if } T_{ij} < t \text{ and } \delta_{ij} = 1 \\ 0 & \text{otherwise} \end{cases}$$

with the intensity

$$\lambda_{ij}(t) = I(t \leq T_{ij})\lambda_0(t)\exp(\alpha_i + \beta Z_{ij}), \tag{A-1}$$

where T_{ij} is the observed time for individual j ($j = 1, 2$) in pair i ($i = 1, ..., n$, like n pairs of twins or matched siblings [1]), δ_{ij} is the indicator of censoring, and α_i and β are unknown parameters.

The Cox's partial log-likelihood is

$$L_n(\beta) = \sum_{i=1}^{n}\sum_{j=1}^{2}\int_0^t \log\left[\frac{\exp\{\beta Z_{ij}\}}{\sum_{j=1}^{2} I(u \leq T_{ij})\exp(\beta Z_{ij})}\right] dN_{ij}(u). \tag{A-2}$$

Using the method in Andersen and Gill [2], Gross and Huber [1] showed that as $n \to \infty$, the Cox estimate $\hat{\beta}$ is consistent for β and $\sqrt{n}(\hat{\beta} - \beta)$ is asymptotically normal. The observed information

$$\sum_{i=1}^{n}\int_0^t I(u \leq T_{i1})I(u \leq T_{i2})\frac{(Z_{i1} - Z_{i2})^{\otimes 2}\exp\hat{\beta}(Z_{i1} + Z_{i2})}{\left\{\sum_j I(u \leq T_{ij})\exp(\hat{\beta}Z_{ij})\right\}^2}\sum_j dN_{ij}(u) \tag{A-3}$$

is consistent for the estimate of the covariance matrix of the Cox estimate $\hat{\beta}$.

If we replace $j = (1, 2)$ by $j = (p, s)$ and rewrite $\lambda_0(t) = h_{0p}(t)$, $\alpha_i = \beta C_i$, $\beta = (\theta, \alpha)$ and

$$Z_{ij}{}^{tr} = \begin{cases} (0, 0) & \text{when } j = p \\ (X_i, 1) & \text{when } j = s \end{cases},$$

the intensity (A-1), Cox's partial log-likelihood (A-2) and observed information (A-3) , respectively, can be rewritten as the models

$$h_{pi}(t) = I(t \leq T_{ip})h_{0p}(t)\exp(\beta C_i) \tag{A-4}$$

$$h_{si}(t) = I(t \leq T_{is})h_{0p}(t)\exp(\theta X_i + \beta C_i + \alpha), \tag{A-5}$$

the log-likelihood

$$l = \sum_{i}^{n} \Delta_{si} \left\{ \theta X_i + \alpha - \log(e^{\theta X_i + \alpha} + S_i) \right\} - \Delta_{pi} \log(1 + P_i e^{\hat{\theta} X_i + \alpha}).$$

and the observed information

$$\sum_{i=1}^{n} e^{\hat{\theta} X_i + \hat{\alpha}} \left\{ \frac{\Delta_{si} S_i}{(e^{\hat{\theta} X_i + \hat{\alpha}} + S_i)^2} + \frac{\Delta_{pi} P_i}{(1 + P_i e^{\hat{\theta} X_i + \hat{\alpha}})^2} \right\} \begin{pmatrix} X_i X_i^{tr} & X_i \\ X_i^{tr} & 1 \end{pmatrix}. \tag{A-6}$$

This means that the estimates $(\hat{\theta}, \hat{\alpha})$ are consistent for (θ, α) and asymptotically normal and the observed information (A-6) is consistent for the estimate of the covariance matrix for $(\hat{\theta}, \hat{\alpha})$. Also this means that $\hat{HR}_{uE} = \exp(\hat{\alpha})$, $\hat{HR}_E = \exp(\widehat{\alpha + \theta})$ and the PERR-ALT estimate

$$\hat{HR}_{PERR-ALT} = \frac{\hat{HR}_E}{\hat{HR}_{uE}} = \frac{\exp(\widehat{\alpha + \theta})}{\exp(\hat{\alpha})}$$

are consistent for $\exp(\alpha)$, $\exp(\alpha + \theta)$ and $\exp(\theta)$ respectively.

Web Appendix B: A simulation study of the bias in the PERR and pairwise methods under the problem of differential case fatality

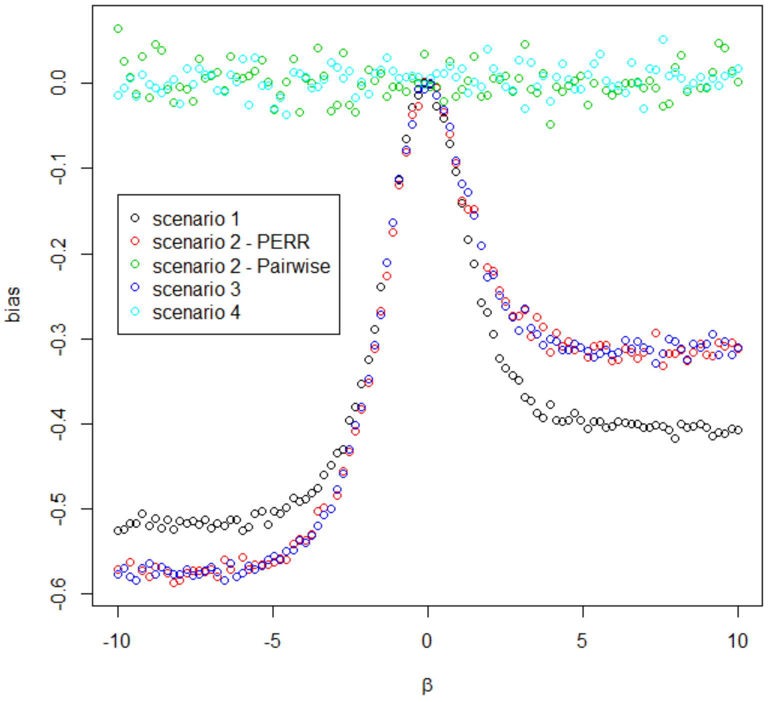

Web figure 1: The performance of PERR and pairwise methods under the problem of differential case fatality.

In this simulation,we generated C from $Bin(1, 0.5)$, X from $Bin(1, 0.3 + 0.4c)$, T_p and T_s from $h_{0p}(t) \exp(\beta c_i)$ and $h_{0p}(t) \exp(\theta x_i + \beta c_i + \alpha)$ respectively with $h_{p0}(t) = \alpha = \theta = 1$ and $\beta \in (-10, 10)$. The sample size was $100,000$ and there was no censoring. We considered the following scenarios:

- **Scenario 1:** No differential case fatality;
- **Scenario 2:** 10% of the subjects with $c = 0$ and 50% of the subjects with $c = 1$ died before study period, x was generated only for the subjects who were alive at the beginning of study;
- **Scenario 3:** No differential case fatality but c was generated from $Bin\left(1, \frac{0.5\times0.5}{0.5\times0.9+0.5\times0.5}\right)$ in place of $Bin(1, 0.5)$;
- **Scenario 4:** All the subjects with $c = 1$ were assumed to have died before the study period and all the subjects with $c = 0$ were assumed to have survived beyond the study period.

Fig 1 shows the biases of the PERR method under these four scenarios and the bias of the pairwise method under Scenario 2. We note that:

- Comparing the bias curves for Scenarios 1 and 2, differential case fatality can not only increase but also decrease the bias of the PERR method;
- The biases of the PERR method in Scenarios 2 and 3 are almost the same. This confirms that the change in the bias of PERR in the case of differential case fatality is in fact the difference between the PERR biases under different distributions of C;
- The results for the pairwise method under Scenario 2 show that the pairwise method is unbiased in the case of differential case fatality;
- The results of Scenario 4 show that the PERR method is unbiased in the extreme case that all the subjects with $c = 1$ died before exposure and all the subjects with $c = 0$ survived beyond exposure.

Web Appendix C: The data for the example in Section 8

References

[1] Gross TS, Huber C. Matched pair experiments: Cox and maximum likelihood estimation. *Scand J Stat* 1987; **14**: 27–41.

[2] Andersen PK, Gill RD. Cox's regression model for counting processes: a large sample study. *The annals of statistics* 1982: 1100–1120.

Web table 1: Data from a longitudinal cohort study of enzyme replacement therapy for patients with Fabry disease

I.d. i	Gender x_5	Age at start of prior period (years) x_{3p}	Prior event t_p (years)	Age at start of study period (years) x_{3s}	Therapy x_1, x_2	Study event t_s (years)
1	Male	27.40	1.22	28.63	B	5.33+
2	Male	44.89	1.11	46.00	B	6.25+
3	Male	65.56	1.71	67.27	B	1.78
4	Male	7.19	0.33	8.53	B	1.63
5	Female	4.01	1.11	8.82	B	0.30
6	Female	13.73	0.84+	14.69	A	0.04
7	Male	31.25	2.29+	34.43	A	1.94
8	Female	45.19	1.43	46.62	A	2.12
9	Male	30.55	0.51	32.74	A	4.08
10	Male	21.64	1.71	23.35	A	1.09
11	Female	45.47	0.33	45.80	A	1.01
12	Female	43.24	1.57	44.81	A	0.86
13	Male	52.29	0.38	52.67	A	3.23+
14	Female	25.17	0.59	27.12	A	5.00+
15	Male	55.36	1.80	58.01	A	0.26
16	Male	65.91	1.11+	67.75	B	0.24
17	Male	56.37	1.08	57.45	A	1.05
18	Female	34.78	0.66	35.44	A	1.05
19	Female	54.55	0.52	55.07	A	1.07
20	Male	57.83	0.35	58.18	A	1.97+
21	Male	33.04	1.15	34.92	A	0.75
22	Male	35.00	0.51	36.07	A	0.52
23	Male	56.67	0.61	57.42	A	1.94
24	Male	58.82	0.50	59.49	A	5.84
25	Male	41.33	2.57	44.70	A	0.48
26	Female	15.85	2.87	19.50	A	0.73
27	Female	27.82	1.02	28.86	A	1.26
28	Male	23.59	3.95+	27.59	A	0.44
29	Male	41.39	4.73	46.22	A	1.53
30	Male	25.48	1.03	26.52	A	2.68
31	Male	35.76	2.97+	39.59	A	2.07
32	Male	55.60	2.36+	58.57	A	1.23
33	Male	54.74	0.68	55.41	B	2.86
34	Male	62.08	2.32+	65.76	B	0.53
35	Male	69.64	1.40	71.69	B	1.02
36	Male	16.41	1.19+	17.59	B	1.03
37	Male	42.72	3.92	46.71	B	3.45
38	Male	49.34	1.83	51.78	B	1.85
39	Male	55.23	1.71	56.94	B	2.05+
40	Male	42.34	0.76+	43.73	B	0.39
41	Male	49.82	0.17	49.99	B	4.83+
42	Male	43.28	1.05	46.90	B	0.69
43	Male	67.26	3.38+	70.75	B	0.90
44	Male	57.66	0.75	58.41	B	6.89+
45	Male	51.72	0.04	51.79	B	0.97+

NOTE:+ indicates censored; no treatment: $x_1 = x_2 = 0$; Therapy A: $x_1 = 1, x_2 = 0$; Therapy B: $x_1 = 0, x_2 = 1$.
